# Microbiome Selection Could Spur Next-Generation Plant Breeding Strategies

**DOI:** 10.3389/fmicb.2016.01971

**Published:** 2016-12-07

**Authors:** Murali Gopal, Alka Gupta

**Affiliations:** Microbiology Section, ICAR-Central Plantation Crops Research InstituteKasaragod, India

**Keywords:** microbiome, holobiont, artificial ecosystem selection, plant breeding, synthetic microbiota

## Abstract

“*No plant is an island too*…”

Plants, though sessile, have developed a unique strategy to counter biotic and abiotic stresses by symbiotically co-evolving with microorganisms and tapping into their genome for this purpose. Soil is the bank of microbial diversity from which a plant selectively sources its microbiome to suit its needs. Besides soil, seeds, which carry the genetic blueprint of plants during trans-generational propagation, are home to diverse microbiota that acts as the principal source of microbial inoculum in crop cultivation. Overall, a plant is ensconced both on the outside and inside with a diverse assemblage of microbiota. Together, the plant genome and the genes of the microbiota that the plant harbors in different plant tissues, i.e., the ‘plant microbiome,’ form the holobiome which is now considered as unit of selection: ‘the holobiont.’ The ‘plant microbiome’ not only helps plants to remain fit but also offers critical genetic variability, hitherto, not employed in the breeding strategy by plant breeders, who traditionally have exploited the genetic variability of the host for developing high yielding or disease tolerant or drought resistant varieties. This fresh knowledge of the microbiome, particularly of the rhizosphere, offering genetic variability to plants, opens up new horizons for breeding that could usher in cultivation of next-generation crops depending less on inorganic inputs, resistant to insect pest and diseases and resilient to climatic perturbations. We surmise, from ever increasing evidences, that plants and their microbial symbionts need to be co-propagated as life-long partners in future strategies for plant breeding. In this perspective, we propose bottom–up approach to co-propagate the co-evolved, the plant along with the target microbiome, through – (i) reciprocal soil transplantation method, or (ii) artificial ecosystem selection method of synthetic microbiome inocula, or (iii) by exploration of microRNA transfer method – for realizing this next-generation plant breeding approach. Our aim, thus, is to bring closer the information accrued through the advanced nucleotide sequencing and bioinformatics in conjunction with conventional culture-dependent isolation method for practical application in plant breeding and overall agriculture.

## The ‘Holobiont’ as Heritable Unit of Selection

In the age of new ecology, the understanding of a plant as no more an individual at its genomic level but a larger genetic entity comprising of its associated microbial genome, ‘the microbiome,’ has given rise to the ‘holobiont’ concept (Zilber-Rosenberg and Rosenberg, 2008; Rosenberg and Zilber-Rosenberg, 2016). A ‘holobiont’ is thus an assemblage of the individual and its symbionts living and functioning as a unit of biological organization (Bordenstein and Theis, 2015; Theis et al., 2016), having the capacity to replicate and pass on its genetic composition; therefore, a unit of selection (Zilber-Rosenberg and Rosenberg, 2008; Booth, 2014; van Opstal and Bordenstein, 2015). The genomic reflection of complex symbiotic interactions of the plant holobiont is governed by its holobiome or hologenome comprising of the host and its microbial genome (Guerrero et al., 2013; Bordenstein and Theis, 2015). In fact, the collective genome of the rhizosphere microbiome is much larger than that of the plant and therefore referred to as the plant’s second genome or pan-genome (Berendsen et al., 2012; Turner et al., 2013). The ‘holobiont’ concept has its roots in the hypothesis that the complex eukaryotic cells have evolved from simple prokaryotes (Embley and Martin, 2006; Douglas, 2014; Koonin and Yutin, 2014). The recent finding of ‘*Lokiarchaeota*,’ a complex archaeabacteria clade that appears to be a missing link between prokaryotes and eukaryotes (Spang et al., 2015), strengthens the presence of prokaryote-to-eukaryote genomic continuum in the plant holobiont (Turner et al., 2013).

## The Microbiome Regulates Holobiont Fitness

**FIGURE 1:**
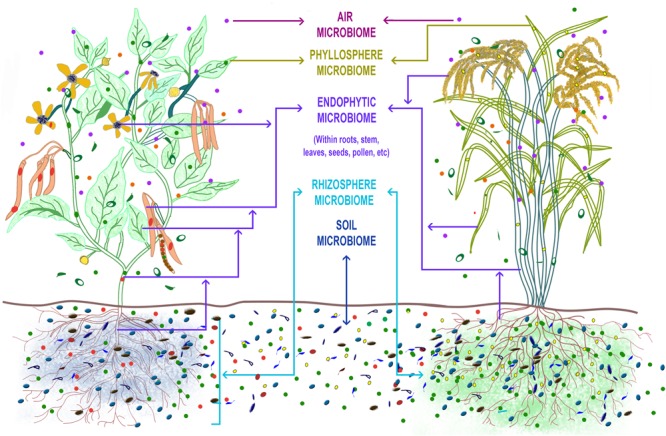
**The ‘Plant Microbiome’ can be described as the sum total of the genomic contribution made by the diverse microbial communities that inhabit the surface and internal tissues of the plant parts.** The rhizosphere, endosphere, phyllosphere constitute the major compartments in which the microbial communities reside in the plant. The soil microbiome is the main source from which the plant selects and builds its microbiome profile. The plant genotype (e.g., dicot bean plant and a monocot rice plant), its root exudates (indicated by blue shade for bean and green for rice), the soil types and properties, and the environmental factors influence the plant microbiome makeup (indicated by different colored microbes inhabiting the plant compartments in bean and rice plant). Mycorrhizal association in both plants is indicated by thin lines extending from the roots into the surrounding soil.

The plant microbiome is compartmentalized into its rhizosphere, endosphere, phyllosphere, and endophytic microbiota (**Figure 1**) with soil largely being the original source of the microbial diversity as observed in *Arabidopsis*, maize, rice, grapevine, cannabis and cucurbits (Bulgarelli et al., 2012; Lundberg et al., 2012; Schlaeppi et al., 2014; Winston et al., 2014; Edwards et al., 2015; Glassner et al., 2015; Zarraonaindia et al., 2015). It has also been reported that the diversity of above ground phyllosphere microbiota includes many taxa that are encountered in soil and water (Vorholt, 2012; Kembel et al., 2014). The selection of the microbes from the soil pool into the plant microbiome is driven by the host (Berg and Smalla, 2009; Hartmann et al., 2009; Hirsch and Mauchline, 2012), modulated by salicylic acid production (Lebeis et al., 2015) as well as phenols (Badri et al., 2013a) released from the roots, and the plant’s evolutionary history (Bouffaud et al., 2014). In the ecological perspective, the plant holobiont and not the plant as an individual, is now known to respond to the various biotic and abiotic perturbations in a given environment. A significant proportion of the plant holobiont’s response is contributed by the microbial symbionts *via* their ecological services of nutrient mineralization and delivery (Terrazas et al., 2016), protection from pests and diseases, and tolerance to abiotic stress. Therefore, the overall fitness of the plant is governed by the self and its microbiota (Vandenkoornhuyse et al., 2015). Several examples where the plant microbiome, particularly of the root and endophytic compartments, has been used to suppress diseases of field and horticultural crops (Mendes et al., 2011; Spence et al., 2014; Cha et al., 2016), improve drought resistance in desert crops (Lau and Lennon, 2012; Marasco et al., 2012) and grapevine (Rolli et al., 2015) and alter above-ground herbivory (Hol et al., 2010; Badri et al., 2013b) have unequivocally proved that the host microbiome indeed impact the fitness of the plants. Next-generation sequencing technologies, advanced bioinformatic analyses coupled with meticulous culture-dependent isolations had been employed in all the above studies to decode the plant microbiome and get to the important bacterial species involved in regulating the phenotypic expression of the plants.

## Microbes Work in Network Mode to Regulate Plant Fitness

A ‘microbiome’ includes bacteria, fungi, actinomycetes, viruses, and protists. However, current information pertaining to the plant microbiome is mostly in reference to the bacterial community. Fungal and virus microbiome research have just begun. Several exciting new studies are unveiling the way in which the plant microbiome performs its duties. They indicate that, like any other species, microorganisms – operate in interlocked networks (van der Heijden and Hartmann, 2016) possessing microbial hubs. Within the networks reside certain keystone species that are critical for the plant-microbe interactions (Agler et al., 2016). It has been found that bacterial communities having high connectence and low nestedness afford them a stabilizing configuration which are able to prevent pathogen attack on some plants (Wei et al., 2015). Before these basic findings became apparent, several works clearly indicated more efficiency when bacteria were applied in a consortium mode for controlling soil borne pathogens (Stockwell et al., 2011; Sarma et al., 2015). Mendes et al. (2011) reported that control of *Rhizoctonia solani* of sugar beet in disease suppressive soil was because of a suite of 111 *Pseudomonas* spp. representing the bulk of antagonistic bacteria isolated from the soil, confirming the results obtained by metagenomic analysis of the disease suppressive soil. Similarly, the work of Koberl et al. (2013) in managing *Ralstonia* disease in medicinal crops in arid ecosystem of Egypt, using a combination of 45 *Bacillus* spp. with *Streptomyces*, highlight the phenomenon that microbes act in network mode. A core consortium of five bacteria was found to rescue tobacco (*Nicotiana attenuate*) from the sudden-wilt caused by *Fusarium–Alternaria* like complex in continuous cropping system (Santhanam et al., 2015). Consortia level application had also helped in improving drought tolerance in grape vine (Rolli et al., 2015) and date palm (Cherif et al., 2015). A combination of *Pseudomonas* spp. altered the post-embryonic root development in *Arabidopsis* that stimulated production of more lateral roots and root hairs and helped the plants perform better under water- and nutrient-limited conditions (Zamioudis et al., 2013). These results indicate that there is better performance of bacteria when they are applied in a consortium underlining their network mode of activity (Hays et al., 2015) in regulating plant fitness. Now, the plant-microbiome relationship via ‘holobiont’ concept is not only restricted to production and protection applications in plants but is also expanding into the realm of plant breeding.

## Conventional Selection Breeding Focused on Plant Genome

Wild plants have evolved over time by selectively assembling plant-beneficial microbiota from soil as their partners. This association was disrupted with the development of agriculture through domestication of important crops. Further disruption entailed as conventional plant breeding and modern genomics-assisted methods focused only on the plant genome, not the hologenome, for developing crops with higher yield, resistance to insect pest and fungal pathogens, tolerance to abiotic stresses such as drought and salinity and characteristics of superior quality for many other desirable attributes. Plant breeding has greatly helped in the food security of the global population. However, domestication of such genetically homogenous crops, cultivated in different ecological conditions, has led to not only the erosion of genetic diversity of the plants; but also extinction of huge microbial diversity in soil that would have been the source of several plant-beneficial microbiota (Perez-Jaramillo et al., 2016).

Domestication and intensive cultivation of a single crop has led to appearance of several qualitative issues such as reduced nutrient use efficiency, increased susceptibility to pests and diseases, inability to overcome abiotic stresses, etc. Domestication also could have removed those traits from plants that were needed to assemble host-specific microbiome affording the plants a very high adaptability to biotic and abiotic stresses (Bulgarelli et al., 2013; Chen et al., 2015). This necessitated application of high quantities of inorganic fertilizer, spraying of insecticides and growth hormones, etc. to maintain the required output (Matson et al., 1997) and on the flip side, drastically losing the soil microbial diversity to a great extent (Weese et al., 2015). Integration of plant-beneficial microorganisms such as nitrogen-fixing bacteria, phosphate solubilizing microbes, plant growth promoting rhizobacteria (PGPR) and arbuscular mycorrhizae were included as agronomic components of crop husbandry and became an environmentally benign alternative to supplement the inorganic inputs. From individual inoculations in the beginning, either bacteria or fungi, to mixed inoculations having both bacteria and fungi yielded desirable results in some crops grown under certain soil and environmental conditions. However, the microbial applications did not always perform to expected levels under different ecological conditions even if the host was the same (Ambrosini et al., 2016). Perhaps singular or combination of two microbes were not able to establish in the soil resulting in below par effectiveness of the bioinoculants. One of the possible reasons could be that the introduced microbes were not able to find their interdependent groups in the foreign soil as in the native soils from which they were originally isolated, which would have helped them to share and exchange critical metabolites like amino acids and sugars to promote their survival under challenging microenvironments. In short, microorganisms are dependent upon their groups for key metabolites to co-occur in an environment having diverse microbial communities (Zelezniak et al., 2015). This again highlights the fact that microorganisms work in network mode and their networking offers a broad base of microbial genomic diversity that could impact plant genetic variability.

## Microbiome Offers Genetic Variability to Plants

Genetic variability in plants, in the form of landraces and wild relatives, is a key factor that conventional plant breeders focused on to produce new varieties and hybrids. This approach, as mentioned earlier, completely focused on the plant genome for the variability. Though, it has yielded splendid results in developing better crops in terms of yield, selection and domestication has led to erosion of plant genetic diversity making plant breeders look for newer sources of variability in plants. With advancement in cutting edge technologies, another new source of variability in plant genetic material *viz.* ‘epigenetics,’ has become a focus in crop improvement programs in recent years (Varshney et al., 2005; Tsaftaris et al., 2008). Epigenetics refers to the different phenotypic manifestations by plants arising from altered expression of genes without any actual changes in the base pairs. Mechanisms driving epigenesis include: DNA methylation, modifications in chromatin *via* modifications in the histones and DNA, and RNA interference. It is considered heritable too. Epigenetics pathways are, therefore, reported to produce phenotypic plasticity in plants which enables them to overcome and reproduce in erratic ecosystems (Pikaard and Scheid, 2014). A report on the recently concluded meeting of Epigenetics of Plants International Consortium in the USA highlighted several themes including basic mechanisms of gene regulation, nucleolar dominance, histone dynamics, DNA methylation, and small RNA functions in plant epigenetics and how they could be used for crop improvement as well as stress and defense response by plants (Slotkin, 2016).

Apart from these, the development of holobiont theory is now unveiling a new basis of genetic variation, which is heritable and offered by the plant microbiome, particularly from the endophytic compartment (Nogales et al., 2016). The dependence of plant on its microbiome is to such a great extent that many plants failed to be cultured as transplants in the absence of bacterial and fungal endophytes (Hardoim et al., 2008). Among the endophytes, seed endophytes are of great importance because seeds not only carry the genetic blueprint of plants during trans-generational propagation, but are home to diverse microbiota too. Advancements in the knowledge of microbiome associated with seeds has, therefore, become critical as it forms the basis of vertical transmission of the microorganisms and hence, acts as a closely linked reservoir of plant endophytic microbiome having many positive impacts on plant germination and growth (Hardoim et al., 2015; Truyens et al., 2015). The transmission of endophytic bacteria can take place from parent plant to seed and then to the seedlings (proper vertical transmission), as in rice, or as in wheat, where bacteria are present in the seed coat, crease tissue and endosperm (Robinson et al., 2016). Studies performed to track the seed microbiome diversity indicated that a core-microbiota of endophytes was conserved during the domestication of wild maize (teosinte) to 10 different varieties of modern cultivated maize (Johnston-Monje and Raizada, 2011). In rice too, about 45% of the bacterial endophytes present in first seed generation were found to be transmitted to the second generation, in a study carried out using PCR-DGGE method with surface sterilized seeds (Hardoim et al., 2012). Bacterial endophytes, such as *Bacillus* spp. transmitted vertically in quinoa, helped in priming of the seeds to counter external reactive oxygen species during germination, thereby, helping the plants to overcome saline and dry soil pressures and improve their stress resistance (Pitzschke, 2016). While terroir was considered as the main source of seed microbial communities (Klaedtke et al., 2015), it was observed that a flux also existed between the rhizosphere and seeds with regard to endophytes. Johnston-Monje and Raizada (2011) have reported such a flux where a seed bacterium, *Enterobacter asburiae*, was found to egress out of the root and colonize the maize rhizosphere, thereby, indicating that seeds can also modulate the rhizosphere microbiome (Johnston-Monje et al., 2016). Thus, in plants like maize, seeds are known to propagate a set of core-microbiome from generation to generation even when grown in ecologically different soil conditions (Johnston-Monje et al., 2014). Seed microbiome, therefore, form an important source of variability in plants.

Next to seeds, the rhizosphere microbiome introduces heterogeneity in plants by affecting their health and productivity (Berendsen et al., 2012; Berg et al., 2014; Pieterse et al., 2016), improving stress tolerance (Rodriguez et al., 2008), and providing an overall adaptive advantage (Haney et al., 2015). The works of Lau and Lennon (2011, 2012), Panke-Buisse et al. (2014), and Wagner et al. (2014) bring to light the role of soil or rhizosphere microbiome in altering the flowering time, indicating the depth of variability microbiomes offer to plant genome. Microbiomes that help plants develop early or late flowering could be used as breeding strategies to escape drought or salinity or heat or cold stress as plants are known to adopt altered flowering time in response to the above abiotic stresses (Kazan and Lyons, 2016). Therefore, sufficient evidence has accrued to show that the microbiome mediates several critical plant functional traits (Friesen et al., 2011), has a great significance on plant phenotypic plasticity (Goh et al., 2013), and can become a new trajectory for plant neodomestication (Duhamel and Vandenkoornhuyse, 2013). In addition to the variability proffered to plants by the microbiome diversity harbored in various plant tissues, another layer of variability is also added by the epigenetic occurrences in the microbiome similar to epigenetic occurrences in plants. DNA methylation in bacteria and archaebacteria not only saves their DNA from self cleavage by its restriction enzymes through restriction modification but is also involved in gene regulation and introduces genetic variability (Casadesus and Low, 2006). Studies using the single molecule real-time (SMRT) sequencing technology in 230 bacterial and archaeal species showed pervasive occurrence of DNA methylation in 93% of the observed species, stressing the incidence of epigenetic events in prokaryotes. The study unraveled twice as many hitherto known DNA binding specificities of methytransferases (MTases) and more than 800 distinct reproducible methylated motifs (Blow et al., 2016). The role of epigenetic events becomes more relevant to our perspective when it is reported to drive the phase change of free-living bacteria such as *Bradyrhizobium diazoefficiens* to symbiotic bacteria because of methylation of specific motifs during the process of symbiosis (Davis-Richardson et al., 2016). Yet another basis of variability in the microbiome is the phenomenon termed as ‘horizontal gene transfer’ (HGT) (de la Cruz and Davies, 2000) that predominantly occurs in rhizosphere environment. This becomes an additional derivative for heterogeneity to the plants. HGT is brought about by the mobile elements such as gene cassettes, plasmids, transposons, and bacteriophages. Thus, it is evident that the microbiome is able to offer important genetic variability to plants that can be considered for future plant breeding strategies, particularly, when an experimental technique such as artificial ecosystem selection is now available to transfer the complete microbial community.

## Artificial Ecosystem Selection of Plant Microbiome

Application of individual microorganism (bacteria or fungi) for improving plant growth, health and overall fitness is comparatively an easy task. But its success in an open system is challenging. Whereas, the application at the microbiome or core-microbiome level has shown to be more successful for the reasons explained elsewhere. However, getting to the relevant bacterial species and preparing their appropriate consortia is the main challenge here because of the complex nature of the microbe-plant interactions. By adopting artificial ecosystem selection method of microbiome transfer (Swenson et al., 2000; De Roy et al., 2014; Voss et al., 2015), strong evidence of heritable changes in drought tolerance in *Arabidopsis thaliana* (Zolla et al., 2013), alteration of flowering time in *Arabidopsis thaliana* genotypes, *Brassica rapa* (Panke-Buisse et al., 2014) and *Boechera stricta* (Wagner et al., 2014) have been reported. The findings of overlapping core-microbiome in sugarcane (Yeoh et al., 2015) and rice (Edwards et al., 2015) with those of *Arabidopsis* (Lundberg et al., 2012) give more hope for cross-compatibility of microbiome transfer with phylogenetically unrelated plant species. Not only bacterial but fungal communities are also shared between different plant compartments, with soil being the main source (Coleman-Derr et al., 2016). Even the important biocontrol fungus *Trichoderma* has been found to have a global core community in endemic plants such as *Aeonium, Diospyros, Hebe, Rhododendron* in comparison with cosmopolitan plants like maize (Zachow et al., 2016).

Interestingly, this new area of synthetic ecology, in which ecologists and medical professionals design beneficial microbial communities, has its origins in almost century-old field ecological studies (Inouye, 2015), such as the one carried out by Henry (1931), wherein control of *Helminthosporium* foot rot disease of wheat was achieved by transplantation of soils suppressive to the pathogen. More recently, using a similar soil inoculation technique, it has been shown that plant communities can be restored quickly on degraded or disturbed land with soil communities such as microbes, nematodes and microarthropods being some of the main drivers (Wubs et al., 2016).

## Host Genome and its Microbiome: Strange, they are Not Bed Fellows Yet in the Strategy for Plant Breeding

As an integral part of the plant hologenome, the plant microbiome is a tool that can be selected together with the plant genome to develop next-generation plant breeding approach. Though some critical views on studies of the microbiome (Hanage, 2014) and hologenome concept (Moran and Sloan, 2015; Douglas and Werren, 2016) exist, it is possible to develop a new plant breeding strategy in which the plant microbiome from a desired field can be developed into a synthetic inoculum and reared with the plant progeny to produce next-generation crops. Challenges for developing large quantities of the microbiome inoculum can be surmounted with the help of next-generation sequencing technologies combined with bioinformatic analyses for determining the pan-microbiome, at different hierarchical scales, on which the plant depends for its fitness (Vandenkoornhuyse et al., 2015) and identifying candidate organisms whose abundance in soil correlates with the plant function (Wagner et al., 2014). Systematic isolations that capture the species present in a community (Bai et al., 2015) which produce the desired phenotypic effect will be able to help kick-start this effort. The proposed new plant breeding strategy is an extension of the bespoke microbiome therapy where the possibility of transfer of core-microbiome from pathogen suppressive soils to pathogen prevalent soils was suggested for managing plant diseases (Gopal et al., 2013). It also draws upon from the ‘neodomestication’ of plants along with its full complement of mutualist theme put forth by Sessitsch and Mitter (2015) as the concept for current century’s agriculture for attaining food security. Berg et al. (2016) advocated integration of plant-associated microbiome in research dealing with plant physiological experiments and breeding approaches for the reason that plant microbiome is known to respond ahead of its host plant to any environmental perturbation, which influences the hormonal activity of the plant and thereby its physiology. This integration would lead to improved understanding of the plant–microbiome interactions and would help in unraveling the functions of the holobiont. They considered it necessary to include cultivar-specific microbiomes in plant breeding studies in view of the high-specificity observed between the symbionts and its host, thus, providing relevant inputs to our proposed perspective on use of microbiome for plant breeding. In another elaborate report, Mueller and Sachs (2015) professed a top–down approach for artificially selecting upon plant and animal microbiomes for improving their health. They described co-evolution as an evolutionary adjustment occurring between two interdependent populations of species in such a way that changes in one population brings about reciprocal changes in the other, and co-propagation as the continuous transmission of host and its microbiome across several generations linking them together in each round of replication. The approaches envisaged by them to establish the functions of microbiome, techniques to manipulate the microbiome through host-mediated selection and to develop starter microbiome culture also form basis of our bottom–up perspective of co-propagating the co-evolved.

## Co-Propagating the Co-Evolved

**FIGURE 2:**
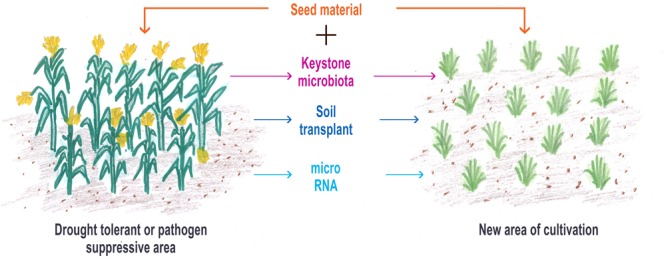
**This represents the direct (keystone microbiota and soil transplant) and the indirect methods (microRNA transfer) of co-propagating the microbiome with planting material from selected situations for raising next-generation crops**.

The approach in our proposed perspective is to co-propagate the co-evolved, i.e., the plant genome and its microbiome. It aims to propel the development in the current knowledge of the microbiome to more practical use in plant breeding, particularly in consideration of disease and drought management, two areas in urgent need of attention to improve agricultural production for food security (Lakshmanan et al., 2014; Haney et al., 2015) in the climate change scenario (Hamilton et al., 2016). Drought and extreme heat, in particular, have been the reason for up to 10% decline in yield of cereals around the world making it the top challenge to crop production (Lesky et al., 2016). Scope for tackling drought using PGPR, i.e., rhizosphere microbiome, is a good option (Ngumbi and Kloepper, 2016). With the current knowledge on the plant microbiome, which is mainly concentrated on bacterial communities, we suggest to co-propagate the microbiome with the plant offspring in the new cultivation with a starter microbiome culture of keystone plant-beneficial microbiota from the target soils. This approach will provide an opportunity to the plants to easily recognize the suite of microbiota with which it had co-evolved and, therefore, preferably recruit them in the new environment. It is also possible that the offspring may have a set of microbiota transferred vertically from the parent, which will enable them to function efficiently in the new environment, if their microbiota are able to interact with the known set of rhizosphere microbiome that was available in the original soil environment in which the parents of the offspring grew. It is now known that the roots attract 2–10 times more types of bacteria than leaves and that the root microbiome is regulated by soil factors such as pH, moisture, and temperature in addition to plant genotype and age (Wagner et al., 2016). Our strategy, therefore, tries to provide the missing microbiome as starter rhizosphere microbiome culture that the plant may require to perform in new environments (**Figure 2**). Providing the starter microbiome culture can be attained either by direct approaches of (i) reciprocal soil transplantation/inoculation from the original soil in which the desired plant had been grown, and (ii) development of synthetic microbiome containing keystone microbiota (plant-beneficial bacteria, arbuscular mycorrhizae, and actinomycetes) or by indirect approach of (iii) transferring microRNA from rhizosphere of target soils to recipient soils. Experiments showing reciprocal soil inoculation or soil transplantation capable of surmounting disease in wheat (Henry, 1931), restore degraded land and giving direction to the type of vegetation grown based on soil inocula (Wubs et al., 2016) and degrade crude oil (Bell et al., 2016) lend credence to the first approach. The work of Calderon et al. (2016) on the restoration of the microbial communities responsible for N-cycling in degraded soil using reciprocal soil inoculum suggests that having an understanding of the priority effects along with the relatedness of the established microbial community and the introduced microbial communities could help in better microbial assemblage and successful restoration of target areas. In a recent work of Bai et al. (2015), it has been shown that, with some meticulous, systematic and exhaustive isolation of bacteria from phyllosphere and rhizosphere, it is possible to capture majority of the species found reproducibly in their respective natural communities. Studies with synthetic communities of bacteria prepared from the isolations could replicate the gnotobiotic reconstitution system allowing for bacterial community establishment. Current research approach for isolation of ‘unculturable’ microbiota from the human gut using cutting edge genomics and bioinformatics tools (Browne et al., 2016) can be followed to isolate keystone microbiota from target soils. More support to the second strategy comes from the work of Panke-Buisse et al. (2016) wherein inoculation of a sub-set of whole microbiome, associated with early flowering in *Arabidopsis thaliana* cultivated on four different types of solid media, was able to reproduce the same flowering timing in *Arabidopsis*. The third strategy mentioned using transfer of rhizosphere microRNA is a possibility of adopting the recent development in human gut microbiology where it has been shown that incorporation of microRNAs harvested from feces is able to restore the disturbed gut microbiome to healthy status (Liu et al., 2016). One recent report by Zhang et al. (2016) highlighting export of microRNAs (miRNA166 and miRNA 159), accumulated in root-hypocotyl junction, cotyledon vasculatures, root tissues, etc. of cotton plants, to the hyphae of pathogenic fungus *Verticillium dahliae* to suppress its virulence, suggests that the third strategy is also feasible.

The ultimate aim of the perspective is to take the research out of the lab and apply it to practical farming techniques using a matching microbiome inoculum to cultivate a given crop. Our perspective reflects the opinion of Denison (2014) who suggested that the key to past and future agriculture depended on increasing the cooperation among plants, their symbionts and the farmers. To make this happen, awareness amongst farmers about the beneficial role of microorganisms in plant production and protection will need to be strengthened through innovative extension programs and communications (Shugart and Racaniello, 2015). Mass-production of the starter microbiome inoculum can be thought of with improvements in the additive printing technology (3D printing technology) of microscopic bacterial communities (Connell et al., 2013). Though the plant microbiome research is in its growing stage, with increased understanding of the mechanisms by which community coalescence takes place *vis-a-vis* the microbial assemblage (Rillig et al., 2016) and several new methods available for studying the rhizosphere environment (Oburger and Schmidt, 2016) including nano-scale tools (Biteen et al., 2016), the challenge can be surmounted with improvement in the knowledge of the microbe-to-microbe and microbe-to-plant interactions by the end of the decade (Mitter et al., 2016) to be able to provide solutions for 21st century crises (Blaser et al., 2016).

## Author Contributions

MG has originally thought about this concept. MG and AG have written the manuscript.

## Conflict of Interest Statement

The authors declare that the research was conducted in the absence of any commercial or financial relationships that could be construed as a potential conflict of interest.
